# Nd:YAG capsulotomy for Ahmed glaucoma drainage implant occlusion by the anterior capsule: a case report

**DOI:** 10.1186/s12886-021-01840-7

**Published:** 2021-02-06

**Authors:** Monica Kenney Ertel, Nathaniel Ryan Gelinas, Taylor John Slingsby, Leonard Keith Seibold, Malik Yaser Kahook, Jeffrey Raymond SooHoo

**Affiliations:** 1 Department of Ophthalmology, University of Colorado, UC Health Sue Anschutz-Rodgers Eye Center, Aurora, Colorado USA; 2 Division of Ophthalmology, Department of Surgery, Denver Health Medical Center, Denver, Colorado USA

**Keywords:** Glaucoma drainage implant (GDI), Tube occlusion, Nd:YAG capsulotomy

## Abstract

**Background:**

Glaucoma drainage implants have been used with increasing frequency for the management of glaucoma. Patients who are candidates for glaucoma drainage devices often have more severe disease and are at risk of vision loss with post-operative elevations in intraocular pressure (IOP). One post-operative complication that can result in IOP elevation after glaucoma drainage device implantation is occlusion of the tube lumen.

**Case presentation:**

Here, we present a novel case of tube occlusion by the anterior capsule in a patient who underwent combined phacoemulsification and Ahmed glaucoma valve implantation. The tube occlusion was successfully managed with Nd:YAG capsulotomy with immediate IOP lowering.

**Conclusions:**

While there have been previous reports of occlusion of the tube lumen by vitreous, iris, blood and fibrin, to our knowledge this is the first report of tube occlusion by the anterior lens capsule and the first report to describe its successful management.

## Background

Glaucoma drainage implants have become an increasingly used surgical approach for the treatment of glaucoma [1]. Glaucoma drainage implants are often reserved for patients with glaucoma that is refractory to medical management and who are at risk for vision loss with post-operative intraocular pressure (IOP) elevations [2]. One potential post-operative complication that can result in persistently elevated IOP after glaucoma drainage device implantation is tube occlusion. Tube occlusion after glaucoma drainage implantation by iris, vitreous, and blood or fibrin clot has been well documented [3–10]. Tube occlusion by the lens capsule is much less common [11, 12]. Here we present a case of Ahmed glaucoma valve (AGV, New World Medical, Rancho Cucamonga, CA) occlusion by the anterior capsule after a combined phacoemulsification and AGV placement. The tube occlusion was relieved with Nd:YAG anterior capsulotomy with a resultant decrease in IOP. We will also review the literature on management of tube occlusions from other, more common, etiologies.

## Case presentation

The patient is a 61-year-old male with a pertinent past medical history of diabetes mellitus who initially presented to our ophthalmology clinic with sudden vision loss in the left eye for four days. At initial visit, his best corrected visual acuity (BCVA) was 20/25 in the right eye and count fingers (CF) at 1 foot in the left eye; pupils were reactive without evidence of an afferent pupillary defect. IOP was 28mmHg in the right eye and 34mmHg in the left eye. His anterior segment examination was normal in both eyes; there was no evidence of neovascularization of the iris. Dilated funduscopic examination revealed a cup to disc ratio of 0.7 in the right eye, with normal macula, vessels and periphery. Left eye dilated examination revealed a cup to disc ratio of 0.5, no evidence of neovascularization of the disc, but there was macular edema, scattered intraretinal hemorrhages in the macula and periphery with tortuosity of the retinal vessels. He was diagnosed with a central retinal vein occlusion and ocular hypertension. He was treated with an intravitreal injection of bevacizumab and started on dorzolamide-timolol in both eyes for his elevated IOP.

At 1-week follow-up, his vision was stable in the right eye and had improved to CF at 5 feet in the left eye. IOP was 16mmHg and 12mmHg in the right and left eye, respectively. His exam was otherwise stable. He was lost to follow-up for 3 months, at which time he presented with left eye pain. At that visit, his BCVA was 20/30 in the right eye, CF at 2 feet in the left eye. IOP was 23mmHg and 42mmHg in the right and left eye, respectively. Anterior segment examination was normal in the right eye. In the left eye, there was microcystic edema of the cornea and a narrow anterior chamber but no evidence of neovascularization of the iris or angle. Gonioscopy revealed no angle structures visible in 270 degrees of the angle bilaterally. Dilated examination was stable in the right eye. Dilated examination of the left eye was also stable with a cup to disc ratio of 0.5 and scattered intraretinal hemorrhages in the macula and periphery and tortuous retinal vasculature. Given the bilateral gonioscopy findings, it was thought that the elevated pressure was potentially secondary to angle closure and a laser peripheral iridotomy (LPI) was attempted in the left eye. IOP after LPI was 28mmHg in the left eye. At 1 week follow-up, the patient was noted to have an IOP of 56mmHg in the left eye with neovascularization of both the iris and angle. An anterior chamber paracentesis and intravitreal bevacizumab injection were performed and the patient was started on oral acetazolamide extended release 500 mg twice daily. After discussion of the risks and benefits, the patient was consented for a combined phacoemulsification with AGV implantation in the left eye. Given the patient’s elevated IOP, evidence of neovascularization and optic nerve cupping, it was felt phacoemulsification alone would not obtain an acceptable IOP.

Intraoperatively, the patient was noted to have extensive posterior synechiae which were lysed with a Kuglen hook and a Malyugin ring (Beye, Wayne, PA) was used for iris expansion due to intraoperative floppy iris syndrome. Nuclear disassembly was unremarkable, and an intraocular lens was placed in the capsular bag. At the conclusion of the phacoemulsification, the main wound was closed with a 10 − 0 nylon suture. AGV placement was then performed in our standard fashion. A traction suture was placed for exposure, followed by a superotemporal conjunctival peritomy and dissection. The superior and lateral rectus muscles were isolated and the plate was sutured 8mm posterior to the limbus and secured using 8 − 0 nylon sutures. The tube was trimmed with a posterior bevel and placed in the ciliary sulcus with the edge of the lumen visible at the pupil margin intraoperatively. Placement of the tube lumen in front of the intraocular lens was confirmed using an anterior chamber cannula to manipulate the lumen. The tube was sutured to the sclera with a 7 − 0 Vicryl suture (Ethicon Inc, Somerville, NJ) and covered with Tutoplast (Katena Products, Inc, Parsippany, NJ) which was secured to the globe with Tisseel fibrin glue (Baxter International Inc., Deerfield, IL). The conjunctiva was reapproximated and secured with Tisseel glue and 8 − 0 Vicryl sutures (Ethicon Inc., Somerville, NJ). Viscoelastic was irrigated from the anterior chamber. The paracentesis wound and conjunctiva were checked and noted to be watertight at the end of the case. The patient was patched and shielded, instructed to stop oral acetazolamide and to follow-up the next day.

**Fig. 1:**
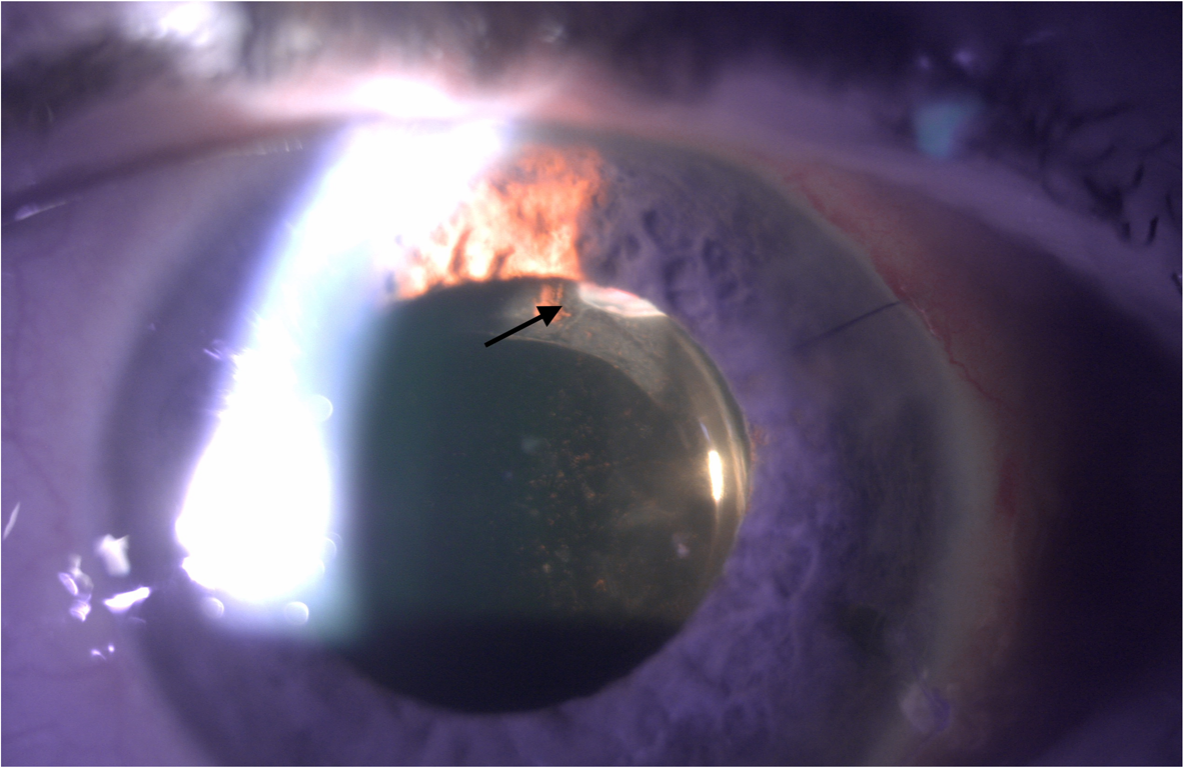
Tube lumen occluded by anterior capsule. This slit lamp photo demonstrates the lumen of the Ahmed glaucoma drainage tube which is obstructed by the anterior capsule (arrow)

**Fig. 2:**
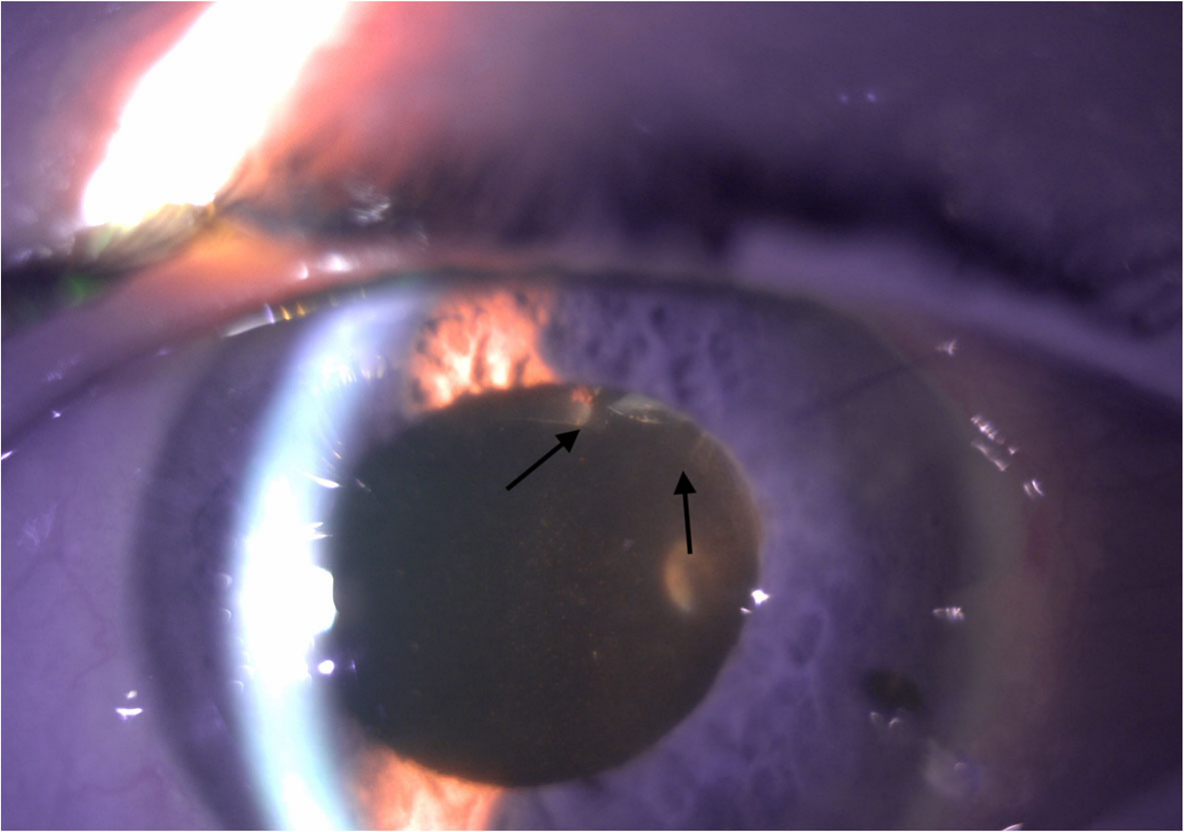
Tube lumen free of anterior capsule after Nd:YAG capsulotomy. This slit lamp photo was taken after Nd:YAG capsulotomy to the anterior capsule. Here, you can see that the tube lumen is free of anterior capsule (arrows, compare to Fig. 1). There was flow noted across the tube lumen after Nd:YAG capsulotomy was performed and a subsequent drop in intraocular pressure

On post-operative day 1, the patient was noted to have stable CF vision in the left eye with an IOP of 45mmHg. Anterior segment examination revealed a deep anterior chamber with clotted blood noted inferotemporally and superotemporally. The conjunctiva over the AGV plate was injected with subconjunctival hemorrhage but was Seidel negative. It was presumed that the lumen of the tube was occluded with either viscoelastic or clotted blood; however, the patient dilated poorly and the lumen of the tube could not be visualized. The patient was restarted on maximum topical medical therapy and asked to follow-up the following day. The patient followed-up three days later, at which point his IOP remained elevated at 50mmHg, although, there was significant medication confusion and patient was admittedly non-adherent. Again, dilation was attempted but did not allow for a view of the tube lumen. The patient was restarted on his IOP lowering drops as well as atropine eye drops. At follow-up the next day, the patient’s IOP remained elevated to 45mmHg. After dilation, the tube lumen was visible at the edge of the pupillary border. The tube was embedded in the lens capsule and the lumen of the tube was noted to be located posterior to and was occluded by the anterior capsule (Fig. 1). After discussion with patient, consent was obtained for Nd:YAG anterior capsulotomy which was performed with the following parameters: 1.6 mJ, 12 shots. During capsulotomy, the lumen obstruction was relieved and flow of aqueous was visible within the tube. After Nd:YAG capsulotomy of the anterior capsule, the tube lumen was free of obstruction and there was an immediate IOP reduction to 6mmHg (Fig. 2). At one-week follow-up after Nd:YAG capsulotomy, IOP remained well controlled at 12mmHg and visual acuity was stable.

## Discussion and conclusions

Glaucoma drainage implant tube occlusion results in decreased drainage across the device and can lead to elevations in IOP. While tube occlusion from a number of different etiologies have been extensively described, such as iris, vitreous and blood/fibrin, there are few reports of tube occlusion by the lens capsule. To date, there are two case reports of Ahmed GDI tube occlusion by the lens capsule, both of which occurred in patients who were aphakic. This is the first case report of tube occlusion by the anterior lens capsule due to placement of the tube lumen in the capsular bag. Here we will review the literature regarding management of tube occlusions by other more common etiologies.

Tube occlusion by vitreous occurs in eyes that have not undergone vitrectomy or in eyes with limited prior vitrectomy. The frequency is also increased with more posterior tube positioning, although it can occur in tubes placed in the ciliary sulcus or anterior chamber due to defects in the posterior capsule or zonular deficiency [3]. Prior reports have demonstrated variable but overall limited utility of Nd:YAG vitreolysis in relieving vitreous tube occlusion [3–5]. Nd:YAG vitreolysis seems to have increased efficacy when the tube lumen has limited occlusion by a sheet of vitreous; in these instances, the vitreous merely covers the opening of the tube lumen and laser vitreolysis can easily relieve the occlusion. When there is more extensive vitreous incarceration in the tube and vitreous plugging the tube, vitrectomy with manual removal of the vitreous plug is necessary to ensure proper tube function [3–5].

Tube occlusions with iris have a similar management strategy as vitreous tube occlusions. Nd:YAG iridectomy of the iris occlusion has been reported, although again success is limited as often times the offending iris is atrophic and floppy and can easily re-occlude the tube lumen [6, 7]. Another approach to managing iris tube occlusion is the use of sling sutures to manually lift the tube off the iris as previously described by Campbell et al. [8] and Katarai et al. [7]. Both reports describe using a 10 − 0 suture (either nylon or polypropylene) thrown through the cornea and around the tube to lift the tube lumen off the offending iris [7, 8]. While these less invasive management strategies are useful in certain situations, more often the definitive treatment is surgical repositioning with removal and reinsertion of the tube.

A third common etiology of tube occlusion is blood or fibrin clot, which can result from inflammation or bleeding that occurs postoperatively. This is particularly true in eyes with neovascular and uveitic glaucoma, both of which are common indications for glaucoma drainage device implantation and predispose patients to blood and fibrin clot formation. With blood/fibrin tube occlusion, intracameral tissue plasminogen activator (tPA) injection has shown good success [10]. The use of tPA to lyse clots has been well established in other organ dysfunction, such as myocardial infarctions, cerebrovascular clots, and pulmonary emboli. A large study by Zalta et al. [9] demonstrated successful clearing or prevention of tube occlusion (in cases which there were blood or fibrin clots in the anterior chamber near the tube lumen but not yet occluding the tube lumen) in 32 of 36 (88.9 %) eyes after glaucoma drainage device implantation with 38.9 % of eyes requiring multiple injections. In eyes that have persistent clogging of the tube with blood or fibrin, surgical intervention with anterior vitrectomy and flushing of the tube may be required to clear the tube lumen and restore function.

In our case, occlusion of the tube was determined to be secondary to the anterior lens capsule. Previous case reports have described occlusion of the tube lumen by the posterior capsule or residual capsule in aphakic patients [11, 12]. However, this is the first case report of tube occlusion due to placement of the tube in the capsular bag during a combination phacoemulsification/Ahmed valve placement. It was decided to place the tube lumen in the ciliary sulcus given reduced risk for corneal complications in this patient with a shallow anterior chamber. Previous reports have demonstrated good IOP reduction with potential for reduced corneal complication with ciliary sulcus placement [13]. While targeting the ciliary sulcus, the lumen of the tube was placed in the capsular bag anterior to the lens but posterior to the anterior capsule. Despite intraoperative confirmation of placement anterior to the lens implant, the lumen of the tube became occluded with the anterior capsule. This highlights the issue of tube length and the importance of consideration of potential occlusion by intraocular structures. While tube revision and anterior chamber placement of the tube is a reasonable option, the minimally invasive nature of anterior capsulotomy made this approach a viable first option.

In conclusion, tube occlusion can occur due to various etiologies, most of which may eventually require surgical intervention to restore tube patency and aqueous flow. Our case report describes tube occlusion by the anterior capsule, which unlike many other cases of tube occlusion, was easily managed with Nd:YAG anterior capsulotomy to clear the lumen.

## Data Availability

All data generated or analyzed during this study are included in this published article.

## Abbreviations

Nd:YAG: Neodymium-doped:yttrium aluminum garnet
IOP: Intraocular pressure
GDI: Glaucoma drainage implant
BCVA: Best corrected visual acuity
CF: Count finger
LPI: Laser peripheral iridotomy
tPA: Tissue plasminogen activator
